# IGF-2 mediated hypoglycemia and the paradox of an apparently benign lesion: a case report & review of the literature

**DOI:** 10.1186/s12902-022-01175-4

**Published:** 2022-10-27

**Authors:** Mairead T. Crowley, Eibhlin Lonergan, Peter O’Callaghan, Caroline M. Joyce, M. Morita, Niamh Conlon, Domhnall J. O’Halloran

**Affiliations:** 1 Department of Endocrinology & Diabetes, Cork University Hospital, Cork, Ireland; 2 Principal Clinical Biochemist, Department of Biochemistry, Cork University Hospital, Cork, Ireland; 3 Department of Internal Medicine, Shimane University Faculty of Medicine, Izumo, Shimane Japan; 4 Department of Histopathology, Cork University Hospital, Cork, Ireland

**Keywords:** Insulin-like growth factor 2, Hypoglycemia, Non-islet cell tumour hypoglycemia

## Abstract

**Background:**

Non-islet cell tumour hypoglycemia (NICTH) is rarely encountered in clinical practice. Insulin-like growth factor 2 (IGF2) is the most common cause of NICTH observed in the setting of mesenchymal and epithelial neoplasia. This is a paraneoplastic syndrome caused by IGF2 activation of the insulin receptor.

**Case presentation:**

An 80 year old female presented with a short history of recurrent episodes of confusion with laboratory confirmed hypoglycemia with a plasma glucose of 2.7 mmol/L on fasting which fulfilled Whipple’s triad. Diagnostic clues to the aetiology at presentation include the fasting pattern of hypoglycemia, hypokalaemia and the absence of weight gain. A 72 hour fast with results showed early hypoglycemia and suppression of serum insulin, c-peptide, and proinsulin. Serum insulin antibody was not detected. Subsequent measurement of the serum IGF2:IGF1 ratio was elevated at 22.3 and consistent with IGF-2 mediated hypoglycemia and imaging studies demonstrated a pelvic mass. Dietary intervention and oral prednisolone abated hypoglycemia prior to surgery. Ultimately, hypoglycemia resolved following operative intervention and steroid therapy was successfully withdrawn. Histopathology was remarkable for dual neoplastic processes with uterine solitary fibrous tumour (SFT) confirmed as the source of IGF2 hypersecretion on IGF-2 immunohistochemistry and a coincidental invasive high grade serous carcinoma involving the fimbria of the right fallopian tube.

**Conclusion:**

The paradox in this case is that the benign solitary fibrous tumour accounted for patient morbidity through secretion of IGF2 and without treatment, posed a mortality risk. This is despite the synchronous presence of a highly malignant fallopian tube neoplasm. This case reinforces the need for thorough clinical evaluation of hypoglycemia to allow prompt and definitive management.

## Background

Hypoglycemia occurring outside of the diabetic population is uncommon. Insulin-like growth factor 2 (IGF-2) mediated hypoglycemia is exceedingly rare in clinical practice [1]. Typically, IGF-2 mediated hypoglycemia is observed in the setting of mesenchymal and epithelial neoplasia [2]. These can represent either benign or malignant processes. Once the condition is recognised and treated in a timely manner, the presence of hypoglycemia in itself does not confer a negative prognosis [2]. It is likely that this disease process represents an underrecognized phenomenon. To our knowledge, this is the first case of IGF2 mediated hypoglycemia in the setting of dual neoplastic processes.

## Case presentation

We describe the case of a previously fit and well 80 year old female who presents with a 10 day history of recurrent episodes of vacancy and confusion. These occurred on a background history of hypertension and primary hypothyroidism. The patient had a limited recollection of these events; however, her husband described transient episodes of unresponsiveness which occurred with increasing frequency in the morning and generally abated following breakfast. Two episodes were associated with low capillary blood glucose readings recorded by paramedics – on the first occasion a capillary glucose of 2.4 mmol/l rose to 5.1 mmol/l following oral intake. A further event coinciding with capillary glucose of 2.4 mmol/l was noted in the emergency department. There was no precipitation of symptoms by exertion or associated weight change. On examination, the patient was clinically well. No clinical features of hypoadrenalism or hypopituitarism were evident. There were no acromegaloid features. The patient had no history of significant alcohol intake, was a lifelong non-smoker and, on collateral history, had no access to oral hypoglycemics or insulin at home.

Whipple’s criteria were fulfilled with a symptomatic episode of confusion with confirmed laboratory serum glucose of 2.7 mmol/L and resolution of symptoms on treatment of hypoglycemia.

Routine admission biochemical investigation demonstrated mild hypokalaemia of 3.0 mmol/l with otherwise normal electrolytes and liver function tests. A synacthen test was normal with a rise in serum cortisol to 554 nmol/L and 697 nmol/L at 30 and 60 minutes respectively. Similarly, anterior pituitary hormone profile was normal including T4 14.4 pmol/L (reference range 9.0–19.1), TSH 3.54 mIU/L (0.35–4.94). Prolactin 341 mU/L (110–562). LH 13.5 IU/L, FSH 32.0 IU/L and oestradiol 58 pmol/L were consistent with post-menopausal status. IGF1 level was low 4.4 nmol/L (4.4–21.8) as was a random growth hormone of 0.26 μg/L. All hormones were measured using Abbott Architect assays except for GH and IGF1 which were measured on the IDS-iSYS analyser. Routine clinical chemistry was performed using the Beckman Coulter AU5800 instrument.

In this context, a prolonged 24 hour fast precipitated early hypoglycemia with plasma glucose of 2.7 mmol/L and a concurrent suppressed serum insulin, c-peptide, proinsulin and 3-hydroxybutyrate. A serum sulphonylurea screen was negative and insulin antibodies were not detected (see Table 1). Investigation proceeded to a 72 hour fast with repeat measurements confirming appropriate suppression of serum insulin and c-peptide in the setting of a symptomatic hypoglycemia of 1.9 mmol/L. In view of these findings, serum was analysed for both IGF-2 and IGF-1 and the IGF2:IGF1 ratio was calculated. IGF2 was analysed by radioimmunoassay and IGF1 was measured using the Mercordia immunoassay.

**Table 1:** Hypoglycemic profiles on prolonged fasting

	24 hr. Fast	72 hr. fast
Plasma glucose	2.7 mmol/L	1.9 mmol/L
Serum insulin^a^	< 10 pmol/L	< 10 pmol/L
Serum proinsulin^a^	< 2.0 pmol/L	< 2.0 pmol/L
Serum c-peptide^a^	< 94 pmol/L	< 94 pmol/L
Serum β-hydroxybutyrate^b^	0.3 mmol/L	0.9 mmol/L
Sulphonylurea screen^a^	Negative	Negative
Anti-insulin antibodies^a^	Negative	–

^a^Analysed in the Peptide Hormones Laboratory, Supra-regional Assay Service, Guildford, Surrey, UK

^b^Analysed in the department of Clinical Chemistry, Sheffield Children’s NHS Trust, United Kingdom (UK)

Results showed a serum IGF-2 of 78.2 nmol/L, IGF-1 of 3.5 nmol/L (4.4–21.8) and an IGF-2:IGF-1 ratio of 22.3 which is consistent with IGF-2 mediated hypoglycemia (see Table 2) [3].

**Table 2:** IGF2 serology profile

	Pre-op	Day 4 post-op	3 months post-op	Reference range
IGF1 (nmol/l)	3.5	5.4	12.6	4.4–21.8
IGF2 (nmol/l)	78.2	57.6	41.7	
IGF2:IGF1	22.3	10.7	3.3	< 10
IGFBP3 (mg/l)	0.8	1.7	3.0	2.0–5.5

**Fig. 1:**
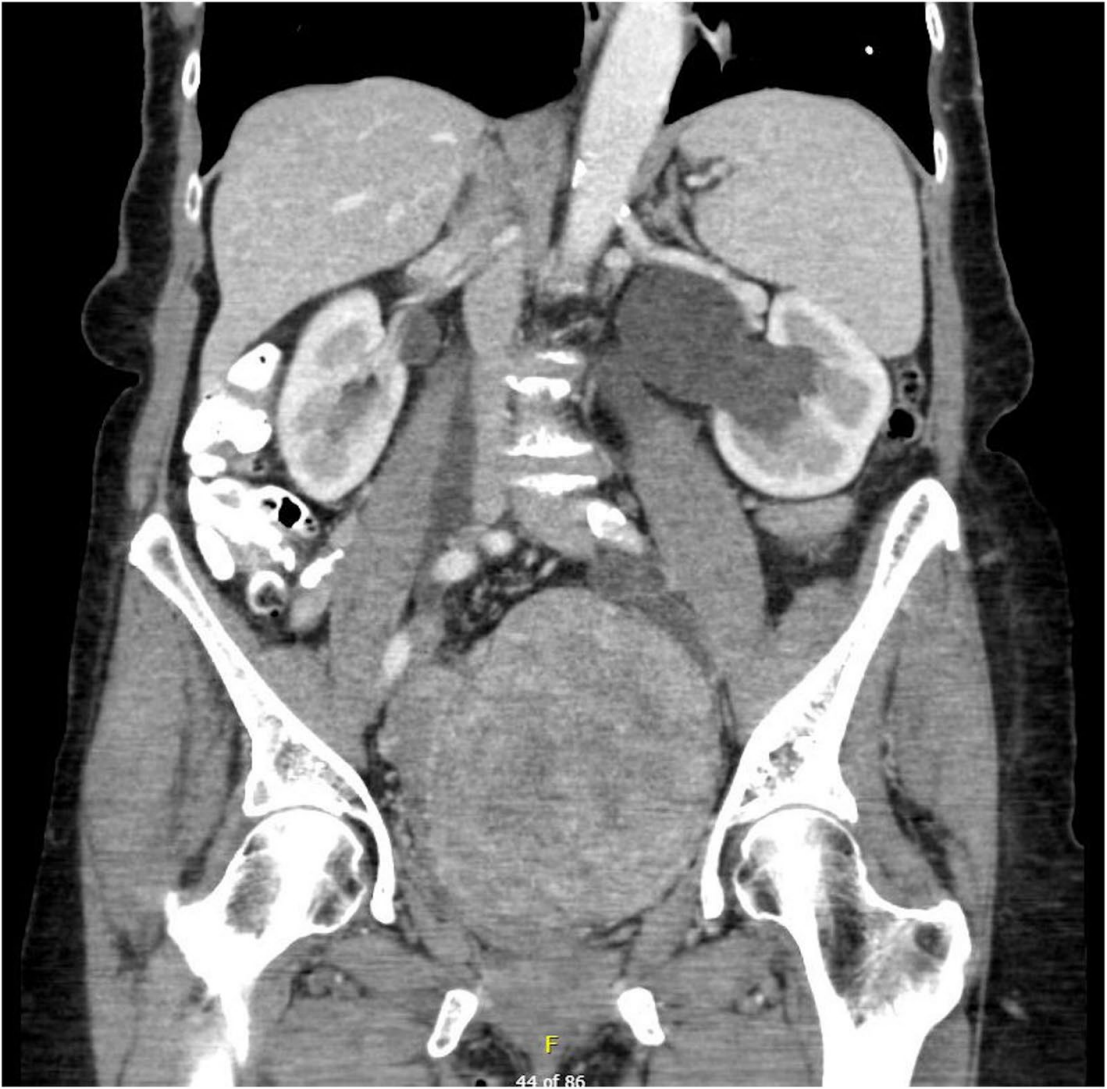
Computed Tomography imaging of pelvic mass

**Fig. 2:**
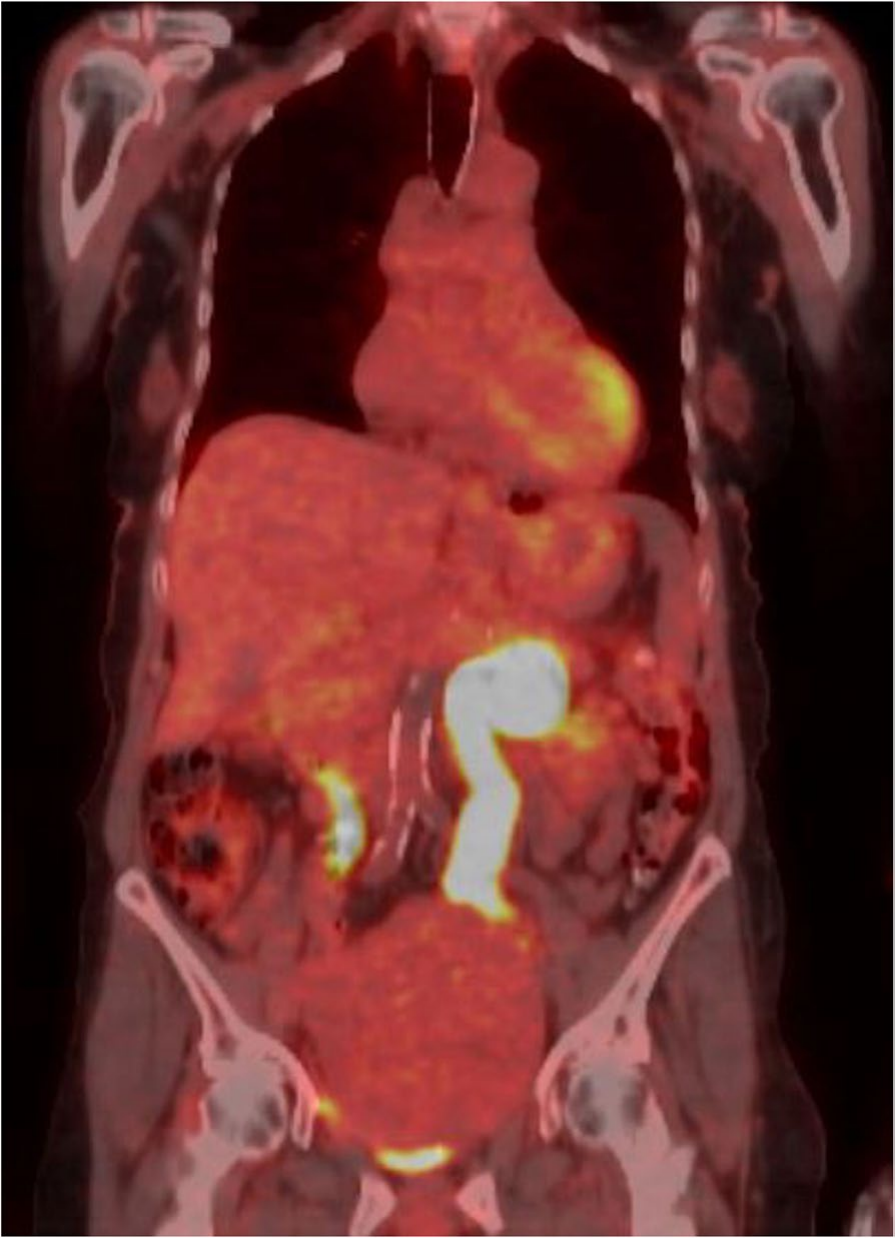
PET-CT imaging of flurorodeoxyglucose uptake

Cross-sectional CT imaging with contrast was performed to investigate for underlying malignancy and revealed a pelvic mass of 13 cm maximal diameter (see Fig. 1). This prompted a follow up fluorodeoxyglucose PET-CT scan which demonstrated mild to moderate uptake of fluorodeoxyglucose by this mass (see Fig. 2).

Clinical nutrition optimised our patient’s intake with frequent high carbohydrate content meals in an effort to minimise hypoglycemia. Despite extensive dietician input, prednisolone 20 mg once daily was required to prevent nocturnal hypoglycaemia while awaiting surgery.

**Fig. 3:**
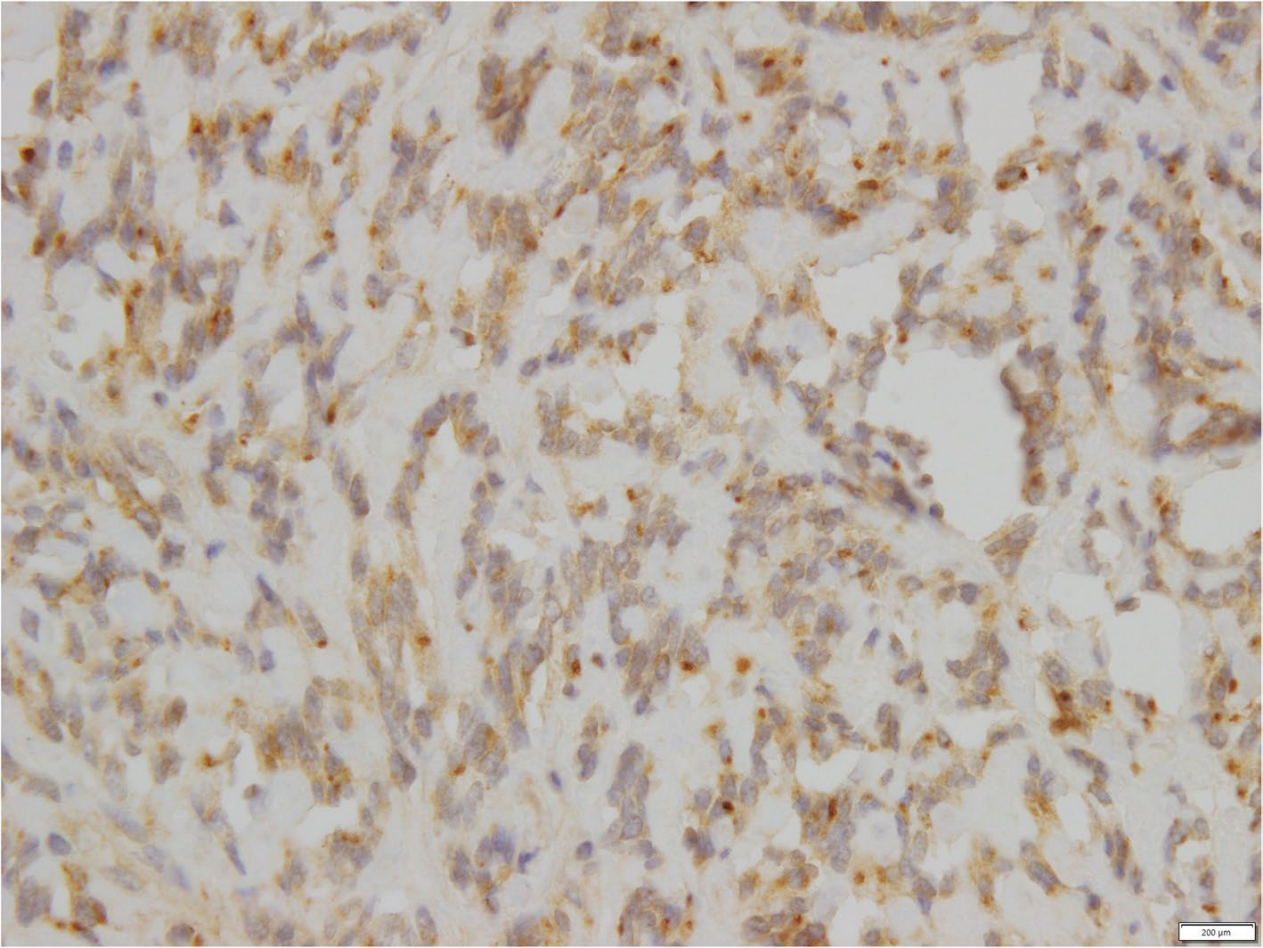
Positive IGF-2 immunostain of fibrous tumour

Our patient was referred to our Gynaecolological Oncology service and proceeded to have a total abdominal hysterectomy and bilateral salpingo-oophorectomy. Intra-operative findings included a 15 cm solid mass arising from the left uterine wall and extending into the broad ligament and left pelvic side wall. The tumour shows classical “pattern-less pattern” SFT spindle cell morphology, without cytological atypia or necrosis. Mitotic count was low (0–1/10 high power field). Immunohistochemistry demonstrated diffuse positivity for STAT6 in tumour cells, consistent with a diagnosis of an SFT. Immunohistochemistry demonstrated that tumour cells were positive for IGF-2 as depicted in Fig. 3 (Anti-IGF2 monoclonal antibody, Merek Millipore, clone S1F2). Incidentally, the right fallopian tube contained a 2 mm invasive high grade serous carcinoma with staging pT1a pNx FIGO classification, stage 1.

At review 3 months post-operatively, our patient reported full symptom resolution. Follow up serology (detailed in Table 3) is indicative of biochemical cure with normalisation of the serum IGF2:IGF1 ratio.

**Table 3:** Classification of the aetiology of hypoglycemia in adults without underlying diabetes [1]

Unwell or medicated individual	Seemingly well individual
1. Drugs Insulin or insulin secretagogues Alcohol Glucagon during endoscopy Quinine Indomethacin 2. Critical illnesses Hepatic, renal or cardiac failure Sepsis (including malaria) 3. Hormone deficiency Cortisol Glucagon and epinephrine 4. Non-islet cell tumour 5. Inborn errors of metabolism	1. Endogenous hyperinsulinism Insulinoma Functional β-cell disorders (nesidioblastosis) Insulin autoimmune hypoglycemia Insulin secretagogue 2. Accidental, surreptitious or malicious hypoglycemia

Table based on Endocrine Society Guideline on Evaluation and Management of Hypoglycemic Disorders [1] and amended to include other causes of hypoglycemia [4]

Chemotherapy for tubal high grade serous carcinoma was completed with 3 cycles of carboplatin. Follow up imaging was reassuring without evidence of disease recurrence. The patient remains clinically well with no further hypoglycemia at 18 months following initial presentation.

## Discussion & conclusions

This case report highlights an unusual and interesting case of hypoglycemia, an uncommon clinical phenomenon outside of the diabetic population. Although it involves one clinical scenario of hypoglycemia, it outlines the systematic approach required to achieve diagnosis and the following discussion highlights pertinent points relating to the diagnosis of pathological hypoglycemia with particular attention to IGF2 mediated processes.

In patients without underlying diabetes, hypoglycemia is diagnosed on the basis of a blood glucose less than 3 mmol/L occurring in the context of Whipple’s triad [1, 5]. This includes symptoms or signs related to hypoglycemia, a laboratory confirmed low blood glucose and improvement of clinical status following treatment of hypoglycemia [1]. Once criteria are fulfilled, investigation as to the cause of hypoglycemia proceeds according to the classification outlined in the Endocrine Society Practice Guideline [1] and detailed in Table 3.

Tumour-induced hypoglycemia is a rare clinical entity encompassing a variety of underlying pathophysiological mechanisms. Eutopic insulin secretion originates from tumours in the pancreatic β-cell but ectopic non-islet cell tumours also occur less commonly [1, 2]. The latter, non-islet cell tumour hypoglycemia (NICTH), encompasses tumour secretion of IGF2, both mature and incompletely processed forms, and less commonly IGF1, somatostatin and glucagon-peptide 1, which stimulate glucose consumption through different mechanisms [2, 6]. The phenomenon of tumour-induced hypoglycemia is observed in cases of significant neoplastic disease as a consequence of increased glucose consumption, liver infiltration and/or pituitary or adrenal gland destruction in the absence of a humoral mediator of hypoglycemia.

The first case of NICTH with IGF2-immunostain positive tumour resection with compatible biochemistry was described in 1988 by Daughaday et al. [7] A 67-year-old presented with recurrent hypoglycemia which resolved after resection of a thoracic leiomyosarcoma with large molecular weight (big) insulin-like growth factor 2 detected on histopathology and in serum. In the interim, further clinical cases with various underlying neoplasm have been described. Traditionally it is believed to be a rarer phenomenon than hypoglycemia due to insulinoma [8]; although this has more recently been challenged due to increased recognition of hypoglycemia in patients with hepatocellular carcinoma [9].

NICTH has been described in a broad range of epithelial and mesenchymal tumours [3]. Hepatocellular carcinoma as reported in the original case description of IGF-2 mediated hypoglycemia in 1929 [8], is the most prevalent neoplasm causing NICTH [3]. Most tumours are large with one review of 78 patients observing tumour diameter of more than 10 cm in 70% [10]. Both benign and malignant pathology is observed [3]. Importantly, hypoglycemia is not a predictor of the size or aggressiveness of the tumour [11].

NICTH is almost exclusively secondary to excessive secretion of IGF-2 or the prohormone, pro-IGF-2. Abnormal activation of promotor regions in the IGF-2 gene, located on the short arm of chromosome 11, results in excessive pro-IGF2 production [3]. Elevated pro-IGF-2 levels oversaturate the prohormone convertase enzyme responsible for conversion of pro IGF-2 to mature IGF-2 [12]. This can lead to a disproportionate rise in big-IGF-2.

Physiologically, IGF-2 forms a 150 kDa complex bound to insulin like growth factor binding protein 3 (IGFBP-3) and acid labile subunit (ALS) [3]. This accounts for 70% of its circulating form with the remaining 30% forming a smaller 50 kDa complex of IGF-2 and IGFBP-3 [3]. Pro-IGF-2 has a lower affinity for binding proteins such as IGF-BP3 and acid labile subunit (ALS). Therefore, big IGF2 forms a lower molecular weight complex. This relatively lower molecular weight results in increased bioavailability of IGF-2 in circulation and increased binding to insulin receptors with subsequent increased peripheral glucose consumption and decreased glycogenolysis, gluconeogenesis and ketogenesis at hypothalamic and hepatic levels [3, 10].

Hypoglycemia generally occurs in the fasting state [13]. Autonomic features such as sweating and tremor, can be blunted in the setting of recurrent episodes of hypoglycemia. Neuroglycopenic symptoms such as confusion, amnesia and seizures tend to predominate [13]. As demonstrated by Jannin et al. in a case series, confusion was the presenting symptom of IGF2-mediated hypoglycemia in three of six patients [14]. This illustrates the importance of recognising hypoglycemia as a reversible cause of confusion and consequent morbidity in the elderly. Hypoglycemia in the setting of IGF-2 excess is associated with increased symptom severity due to lack of ketogenesis as activation of the insulin receptor by IGF-2 not only stimulates glucose utilisation but also suppresses free fatty acid release therefore limiting brain energy supply [15]. NICTH should be considered in any patient with an established oncological diagnosis as well as cerebral metastasis, opioid use and infection which can mimic a presentation of hypoglycemia [16].

Hypokalaemia is a feature in approximately half of all cases at presentation, as was the case with our patient, however is generally only recognised in retrospect [10]. The mechanism for this is not established. Acromegaloid features as a consequence of IGF receptor activation have been described in the context of a pelvic clear cell sarcoma [17]. The woman presented with skin tags and course facial features which subsequently settled after tumour resection. These features are hypothesized to arise from IGF-2-mediated stimulation of insulin-related and IGF-1-related receptors [3].

Initial evaluation for the presence of Whipple’s triad is essential. Hypoglycemia can be the presenting feature of hypopituitarism and hypoadrenalism as well as acute presentations of hepatic or renal failure, sepsis and starvation [18]. A clinical review, including thorough history and examination to elucidate features of systemic disease and medication use completes initial work up. Biochemical assessment of glucose, insulin, c-peptide, proinsulin and β-hydroxybutyrate during a hypoglycemic episode will differentiate pathologies [1]. The hypoglycemic event can be spontaneous or provoked by a prolonged supervised fast of up to 72 hours or a mixed meal test.

NICTH is potentially misdiagnosed or underdiagnosed due to its rarity, non-classical clinical presentation and potentially ambiguous biochemical profile [19]. IGF-2-mediated hypoglycemia, the main aetiology of NICTH, involves the secretion of both mature IGF2 and incompletely processed forms, namely ‘big IGF2’ [3]. The timing of the clinical detection of hypoglycemia in the setting of an IGF-2-oma is variable. Initial tumour presentation with hypoglycemia was observed in 48% of patients in a retrospective review, as was the case with our patient, while in the remaining 52%, tumour detection preceded the recognition of hypoglycemia [10].

Patients with IGF-2 mediated hypoglycemia manifest low serum insulin, proinsulin, c-peptide and B-hydroxybutyrate during hypoglycemia [10]. Serum IGF-1 and GH levels are low which differs from other causes of hypoglycemia which generally precipitate an increase in serum GH [10, 20]. Measurement of serum IGF-2 levels in isolation is of limited diagnostic value as a normal value does not exclude diagnosis [21]. In a review, 58% (18/31) of serum IGF2 measurements in patients with IGF2-producing NICTH were within normal reference range [10]. Concurrent measurement of serum IGF-1 allows calculation of a ratio of IGF-2 to IGF-1. In health, a normal ratio is 3:1. In the setting of recurrent hypoglycemia, a molar ratio of greater than 10 confirms NICTH [21]. Of note, false positive ratio elevation can be seen in the setting of malnutrition and sepsis but individual IGF-1 and IGF-2 values are below reference range [22]. Furthermore, IGF-2/IGF-1 ratio is influenced by IGFBP-3 and therefore renal failure, through a reduction of IGFBP-3 levels, can result in a false negative IGF-2/IGF-1 ratio [3].

A review of the profile of circulating glucose-regulatory hormone in 37 patients with big IGF2-mediated hypoglycemia reported lower random growth hormone level during hypoglycemic episodes and suggested that this may be a useful indicator for the presence of IGF-2 producing NICTH [23]. Plasma cortisol levels when measured during hypoglycemia were significantly reduced in this group also. The pathophysiology for this is not clearly understood. It is suggested that this may be related to the attenuation of counter-regulatory hormonal response to repeated hypoglycemia as observed in the diabetic and insulinoma cohort [23].

Initial treatment of hypoglycemia involves oral and potentially parenteral glucose therapy. Once hypoglycemia is corrected in the acute sense, further episodes are avoided through glucocorticoid therapy [24]. Steroids increase hepatic gluconeogenesis, inhibit peripheral uptake of glucose and promote lipolysis but use is limited by an ongoing dose titration requirement. Glucocorticoid effectiveness is only achieved when steroid dose exceeds a patient specific threshold and reduction or withdrawal of therapy can lead to recurrence of hypoglycemia [24]. Diazoxide has been successfully used in the short term to stimulate hepatic glycogenolysis [25]. Glucagon infusion pumps are also efficacious however use is limited to the shorter term [25]. Recombinant growth hormone (rGH) is a useful agent for glucocorticoid-refractory cases not amenable to operative intervention [26]. It increases gluconeogenesis and peripheral glucose uptake as well as increasing IGFBP-3. Large 150 kDa complexes of IGF2 bound to IGFBP3 reduce the bioavailability of IGF2. Somatostatin therapy has not been effective in controlling hypoglycemia in the context of IGF2-mediated process [27]. Although reports have suggested that inhibition of the mammalian target of rapamycin (mTOR) pathway improves hypoglycemia associated with malignant insulinoma, it has not been replicated in IGF2 mediated hypoglycemia [28]. Surgery with the goal of complete tumour resection is necessary to alleviate hypoglycemia in the long term [2]. Debulking is considered where complete surgical excision is not feasible. Neoadjuvant chemoradiation and arterial embolization has been described in the literature in the context of a non-resectable solitary fibrous tumour [29].

In conclusion, this case demonstrates the caveats in achieving a diagnosis of IGF-2 mediated hypoglycemia and highlights a novel case involving two separate neoplastic processes. Although an incidental finding of serous carcinoma was made, the paradox in this case is that the apparently benign solitary fibrous tumour accounted for the patient’s morbidity, through secretion of IGF2 and without treatment, posed a significant mortality risk.

## Data Availability

Data to support the findings in this case report can be requested from the corresponding author.

## Abbreviations

IGF2: Insulin-like growth factor 2
IGF1: Insulin-like growth factor 1
CT: Computed tomography
PET-CT: Positron emission tomography-computed tomography
NICTH: Non-islet cell tumour hypoglycemia
SFT: Solitary fibrous tumour
rGH: recombinant growth hormone
